# Integrated pathological analysis to develop a Gal-9 based immune survival stratification to predict the outcome of lung large cell neuroendocrine carcinoma and its usefulness in immunotherapy

**DOI:** 10.7150/ijbs.76936

**Published:** 2022-09-25

**Authors:** Yun Che, Zhiwen Luo, Yanan Cao, Jingnan Wang, Qi Xue, Nan Sun, Jie He

**Affiliations:** 1 Department of Thoracic Surgery, National Cancer Center/National Clinical Research Center for Cancer/Cancer Hospital, Chinese Academy of Medical Sciences and Peking Union Medical College, Beijing, 100021, China.; 2 Department of Hepatobiliary Surgery, National Cancer Center/National Clinical Research Center for Cancer/Cancer Hospital, Chinese Academy of Medical Sciences and Peking Union Medical College, Beijing, 100021, China.; 3 Pathology Department, National Cancer Center/National Clinical Research Center for Cancer/Cancer Hospital, Chinese Academy of Medical Sciences and Peking Union Medical College, Beijing, China.; 4 Department of Radiation Oncology, National Cancer Center/National Clinical Research Center for Cancer/Cancer Hospital, Chinese Academy of Medical Sciences (CAMS) and Peking Union Medical College (PUMC), Beijing, China.

**Keywords:** large cell neuroendocrine carcinoma, galectin-9, prognosis, biomarker, immunotherapy, tumor-infiltrating lymphocytes, immune suppression

## Abstract

This study aimed to integrate the cell spatial organization to develop a Gal-9-based immune survival stratification in the lung large cell neuroendocrine carcinoma (LCNEC) and investigate its potentials to immunotherapy.

The expression of Gal-9 and other twelve immune markers were evaluated in 122 cases of surgical LCNEC samples from our center using immunohistochemistry. The Gal-9-based immune survival stratification risk score was constructed and its predictive performance was evaluated. Then, we thoroughly explored the effects of Gal-9 and immune risk score on LCNEC immune pathways, immune micro-environment and immunotherapy sensitivity in different cohort and platform, and made a validation in pathology images using Histology-based Digital-Staining (HDS).

In 122 LCNEC samples, 43 cases were positive Gal-9 expression on tumor cells (Gal-9 TC). Increased Gal-9 TC predicted worse overall survival. Gal-9's interaction with other immune markers added to the immune suppression and immune tolerance in LCNEC. Immune protein marker-based risk score consisting of Gal-9, CD3, CD4, PD-L1, and PD-1 was developed and validated to robustly discriminate survival high-risk or low-risk in LCNEC patients. The high-risk group characterized by immune-desert tumor had less various T cells. The low-risk group featuring immune-inflamed tumor was more likely to respond to anti-PD1 immunotherapy. HDS in 122 LCNEC samples' 108,369 cells validated that the high-risk group had more tumor cells, less stromal cells, less lymphocytes, higher tumor cell nucleic solidity and lower stromal cells nucleic solidity.

An integrated pathological analysis confirms the Gal-9 based immune survival stratification is distinctively related to micro-environment status involved in immune suppression and immune tolerance and could act as a combinatorial biomarker to predict the outcome of LCNEC. These findings may help effectively stratify LCNEC patients sensitive to immunotherapy.

## Background

Lung cancer is associated with significant morbidity and mortality 1. The World Health Organization classifies pulmonary neuroendocrine tumors as typical carcinoids, atypical carcinoids, large cell neuroendocrine tumors (LCNEC), and small cell lung cancer (SCLC). LCNEC are rare, accounting for 2-3% of all lung cancers 2. However, LCNEC are more difficult to treat compared to non-small lung cancer. Surgery is the mainstage of treatment for early-stage LCNEC. NSCLC-based treatments may perform better than traditional platinum-etoposide regimens in the management of LCNEC 3. The combination of immunotherapy and chemotherapy plays an important role in lung cancers 4,5. However, the role of immune checkpoint inhibitors (ICIs) in LCNEC remains unclear.

Galectin-9 (Gal-9) was first implicated in Hodgkin's lymphoma 6. Gal-9 is an immunological TIM-3 ligand that modulates cell-cell and cell-pathogen interactions in the tumor micro-environment 7. The binding of Gal-9 combined with TIM-3 induces T cell exhaustion and impedes the activity of natural killer (NK) cells 8,9. Gal-9 promotes myeloid-derived suppressor cells induction and results in myeloid cell-mediated T cell suppression 10,11. Nevertheless, the prognostic role of Gal-9 in LCNEC remains unclear.

Due to its rarity, only a few prognostic markers for LCNEC have been reported 12,13. However, no immune markers associated with the morphological characteristics of LCNEC have been identified. Histo-pathological assessment provides valuable information on tumor grade and immune infiltration. Intra-tumoral heterogeneity reflects morphological diversity within cell populations. Computer-extracted features associated with tumor cellular diversity might affect prognosis. Several studies have attempted to develop computational pathology-based prognostic models for lung cancer 14,15.

In this study, we used integrative pathological assessment to develop and validate an immune marker-based risk panel including Gal-9 as a predictor of survival in patients with LCNEC. Bioinformatics and histology-based digital-staining (HDS) were then used to investigate the effects of the risk score on the immune micro-environment, immunotherapy sensitivity and tumor morphology.

## Methods

### Patients

The present study retrospectively enrolled 122 patients with LCNEC assessed at the Cancer Hospital, Chinese Academy of Medical Sciences (CICAMS) from 2012-2019. Total 122 cases of patients with a pathological diagnosis of LCNEC treated with surgery were enrolled. These patients with a pathological diagnosis of stage I-III LCNEC were included. Patients were treated with surgery only or surgery combined with adjuvant chemotherapy. The adjuvant treatment options mainly include SCLC-type regimen (the combination of cisplatin-etoposide) and NSCLS-type regimen (the cisplatin-based chemotherapy). The LCNEC patients who received preoperative neoadjuvant therapy or non-surgical therapy were not included in this study. Two independent reviewers screened the histo-pathological characteristics of the tumor samples. All patients provided written informed consent for research participation.

### Hematoxylin & eosin staining

Hematoxylin & eosin (HE) staining was performed based on standard procedures. The mounted sections were then examined and photographed using a LEICA DM 3000 LED microscope (Weztlar, Germany) and analyzed using the Image-Pro Plus 6.0 software.

### Immunohistochemistry for galectin-9 and other twelve immune molecule markers

All formalin-fixed, paraffin-embedded tissue sections were rinsed with distilled water after dewaxing with xylene and alcohol. The target retrieval solution kit (DM828 or DM829, DAKO) was then used for antigen repairing, and 3% hydrogen peroxide was used to reduce the background staining. The primary antibody was incubated overnight, and the secondary antibody (EnVisionTM Detection Kit, DAKO) was incubated at room temperature for 30 min, followed with DAB staining. The staining intensity was examined using the LEICA DM 3000 LED microscope. The primary antibodies required were: Gal-9 (54330, CST), FOXP3 (12653, CST), PD-L2 (82723, CST), OX40L (203220, Abcam), CD4 (UMAB64, ORIGENE), CD8 (SP16, ORIGENE), CD3 (SP7, MXB), PD-1 (MX033, MXB), and PD-L1 (22C3, DAKO). Using the PANNORAMIC slide scanner to put the tissue slide on the machine, the slide was gradually moved under the lens of the scanner for imaging, with tissue information on the slide imaged to form a folder. The CaseViewer2.4 software was used to magnify the image 1-400 times. The Classifier module of the Halo v3.0.311.314 analysis software was used to distinguish between tumors and stroma. The Indica Labs - Multiplex IHC v2.2.0 module in the Halo v3.0.311.314 analysis software was used to quantify the number of positive cells, the total number of cells, and the tissue area (mm²) in the target area of each section. The positive cell rate (%) and the density of positive cells (cells/mm²) were also calculated.

### Immunohistochemistry cut-off value

Gal-9, PD-L2, OX40L, and PD-L1 staining of ≥1% tumor cells (TC) or tumor infiltrating lymphocytes (TILs) was considered positive. CD3, CD4, CD8, PD-1 and FOXP3 staining on ≥1% TILs were considered positive. Stained sections were reviewed separately by two independent reviewers blinded to the clinical data. The optimal cut-off values of all staining markers were calculated using survival analysis.

### Clinical value of the galectin-9-based risk score model in patients with LCNEC

We reviewed and stained all HE slides from 122 cases of LCNEC to collect comprehensive clinic-pathological data, with emphasis on established prognostic markers described for cancer prognosis, such as the spreading through air spaces (STAS) and Ki-67.

### Repeated lasso and risk score models

The 122 patients were randomly divided into the training and test cohorts at a ratio of 7:3. We performed univariate analyses and log-rank test to identify the immune-related proteins associated with prognosis in the training cohort (N=85). For genes associated with prognosis, a Cox proportional hazards model (iteration = 2000) with a lasso penalty was used to find the best-fitting immune survival stratification model utilizing the R package “glmnet”. The best-fitting model was used to determine the immune marker-based risk score. Then, the concordance (c)-index, the AUC of receiver operating characteristic curve (ROC), and Kaplan-Meier curve were applied to validate the predictive ability of the model in a dependent testing dataset, by using the “survcomp”, and “timeROC” R package. A model with a larger c-index or AUC represents a better prediction ability.

### RNA microarray validation: the role of LGALS9 mRNA expression in lung neuroendocrine tumors

The Gene Expression Omnibus (GEO) Database was used to verify the expression of Lgals9 mRNA in LCNEC tumor tissues. According to WHO 2015 classification, lung neuroendocrine carcinomas represent high-grade forms, comprising LCNEC and SCLC, while lung carcinoids are low-grade forms. Based on the following inclusion criteria, GEO mRNA expression were identified: mRNA sequencing from lung neuroendocrine tumor (LCNEC, Lung Carcinoids, and SCLC) patients with survival data, complete mRNA expression data. The exclusion criteria were as follows: mRNA expression could not be compared due to the insufficient data, mRNA sequencing not from humam tumor tissues. GEO expression profiling was then downloaded for screening differentiating genes (DEGs) between groups using Wilcoxon test, and Kaplan-Meier curve was adopted for testing the prognosis of different groups.

### Gene set enrichment analysis (GSEA)

For the purpose of exploring different biological pathways associated with Gal-9 expression, the “clusterProfiler” R package was used to perform GSEA. All GSEA parameters were left at their default settings and the GEO mRNA expression dataset was divided into two groups according to Gal-9 expression levels. Gal-9-related genes in top immune GSEA pathways were identified, and the relationships between Gal-9 and these genes were visualized by STRING software (version 11.5, https://string-db.org/).

### The landscape of immune infiltration in patients with LCNEC

Immune cell abundance identifier (ImmuCellAI) analysis was used to assess immune infiltration in patients with LCNEC. ImmuCellAI is an online database for immune infiltration analysis that estimates 24 immune cells' abundance from gene expression dataset, in which the 24 immune cells are comprised of 18 T-cell subtypes and 6 other immune cells: B cell, NK cell, Monocyte cell, Macrophage cell, Neutrophil cell and DC cell. The difference of immune infiltration landscapes between different levels of LGALS9 alone, and the association of the combinational level of CD4, CD3E, LGALS9, CD274, and PDCD1 with immune infiltration were revealed comprehensively. Importantly, when investigating our protein-based model in an RNA dataset, we set a stringent criteria to avoid the influence of data variation: 1. The low risk level has positive RNA expression of both CD4 and CD3E of which the coefficient factors in our protein-based model are negative numbers, meanwhile, has no more than 2 of positive LGALS9, CD274, and PDCD1 of which the coefficient factors in our protein-based model are positive numbers; 2. The high risk level has both negative RNA expression of CD4 and CD3, meanwhile, at least 1 of positive LGALS9, CD274, and PDCD1.

### Putative Immunotherapy response in patients with LCNEC

According to the previous description, we used subclass mapping analysis to predict immune checkpoint blockade responses in the High- and Low-risk groups 16.

### Deep learning of histopathology images at the single cell level

It is demonstrated that the image-derived tumor micro-environment (TME) features, such as the spatial organization of different cell types and single cell characteristics could be linked to the gene expression of biological pathways. In order to further investigate the pathological phenotypes in our 122 LCNEC patients with different survival risk level stratified by our Gal-9 based immune survival stratification, we segmented the tumor, stroma, lymphocyte, macrophage, karyorrhexis, and red blood cell nuclei from their standard H&E-stained pathology images by Histology-based Digital-Staining (HDS), a deep learning-based computation model, which could classify eight cell nuclei features that characterize the TME. The difference of image-derived TME features between low- and high-risk group was investigated.

### Statistical analysis

The correlations between Gal-9 expression, clinical factors, and immune-related protein markers were evaluated using a Chi-squared tests. Cox regression and Kaplan-Meier survival curves were used to compare outcomes between different patient groups. We calculated the overall survival (OS) from the date of surgery to the end of follow-up or death. All statistical tests were two-sided, and a p-value <0.05 was deemed statistically significant. The best cut-off values for continuous numerical variables were calculated using the R “survminer” package. Random seed is 941022. The Microsoft Excel (Version 2016), SPSS (Version 25.0) R Studio (Version 3.6.1) software packages were used for data analysis.

## Results

### Patient features

The study sample comprised 122 patients, the majority (n = 91, 74.6%) of whom were diagnosed with pure LCNEC, while 31 patients (25.4%) were mixed with other types. The sample consisted primarily of males (n = 112, 91.8%) and most (n = 110, 90.2%) patients were non-smokers. The majority (n = 81, 66.4%) of patients had stage I-II LCNEC (Table 1).

### Galectin-9 expression, clinical factors and immune parameters

**Figure 1:**
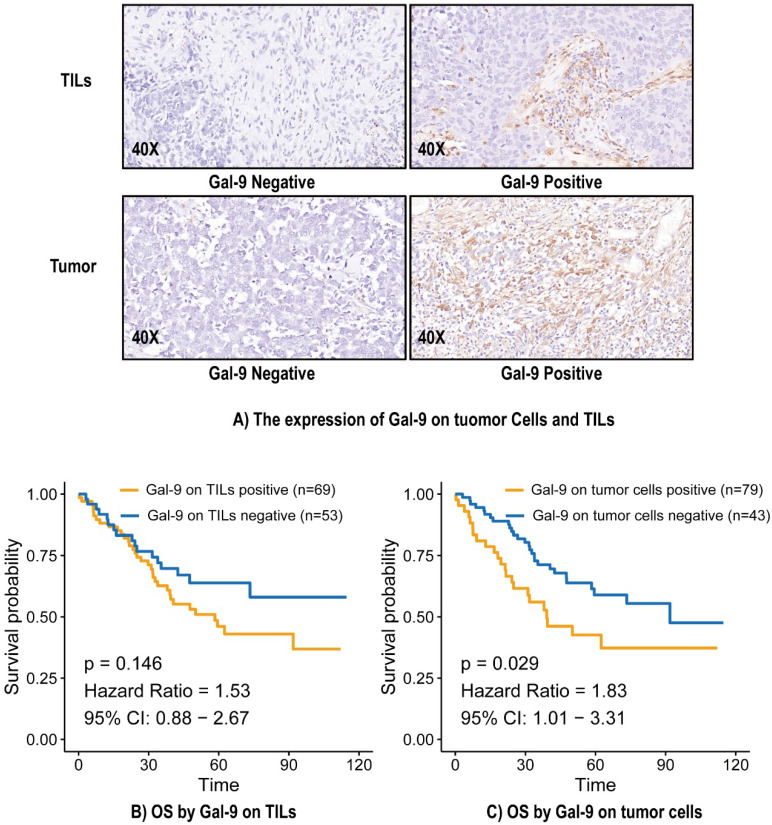
The expression of Gal-9 on tumor cells and TILs (A) and survival analysis (B and C). Gal-9, galectin-9; IHC, immunohistochemistry; TILs, tumor-infiltration lymphocytes; OS, overall survival.

In total, 43 (35.2%) cases were positive for Gal-9 expression on tumor cells, and 69 (56.6%) cases were positive for Gal-9 expression on TILs (Figure 1A). The level of Gal-9 expression on tumor cells was significantly correlated with pleural invasion (P = 0.007) (Table S1). Gal-9 expression on tumor cells was significantly correlated with CD4 on TILs (P = 0.03), CD8 on TILs (P = 0.03), and PD-L2 (P = 0.02) on tumor cells. Gal-9 expression on TILs was significantly correlated with CD4 on TILs (P = 0.025), CD8 on TILs (P = 0.001), FOXP3 on TILs (P = 0.022), OX40L on tumor cells (P = 0.03), OX40L on TILs (P = 0.00018), PD-1 on TILs (P = 0.002), PD-L1 on tumor cells (P = 0.026), and PD-L2 on tumor cells (P = 0.048) (Table S2).

### Relationship between Gal-9 status and prognosis in LCNEC

The median overall survival (OS) was 38.95 months, and 51 (41.8%) patients had died by the end of the study period. The median OS calculated using the Kaplan-Meier method was 73.0 months. There was no significant relationship between Gal-9 expression on TILs and OS (Hazard Ratio [HR] = 1.53, 95% Confidence Interval (CI): 0.88-2.67, P = 0.146) (Figure 1B). Gal-9 positivity on tumor cells predicted poorer OS (HR = 1.83, 95% CI: 1.01-3.31, P = 0.029) (Figure 1C). A subgroup analysis based on Gal-9 expression levels on tumor cells was also performed. The predictive abilities of Gal-9 expression levels on tumor cells were more pronounced when the status of various other immune markers were taken into consideration, particularly CD4 negativity (HR = 3.65, 95% CI: 1.06-6.25, P = 0.037), OX40L TC negativity (HR = 3.05, 95% CI: 1.04-5.05, P = 0.04), OX40L TIL positivity (HR = 2.74, 95% CI: 1.17-4.3, P = 0.015), PD-1 positivity (HR = 2.16, 95% CI: 1.02-3.31, P = 0.044), and PD-L1 TIL positivity (HR = 4.75, 95% CI: 1.1-8.41, P = 0.033) (Figure S1). It indicated the good performance of Gal-9 on tumor cells in predicting the OS in LCNEC independently.

### Construction and validation of galectin-9 based immune survival stratification risk score

**Figure 2:**
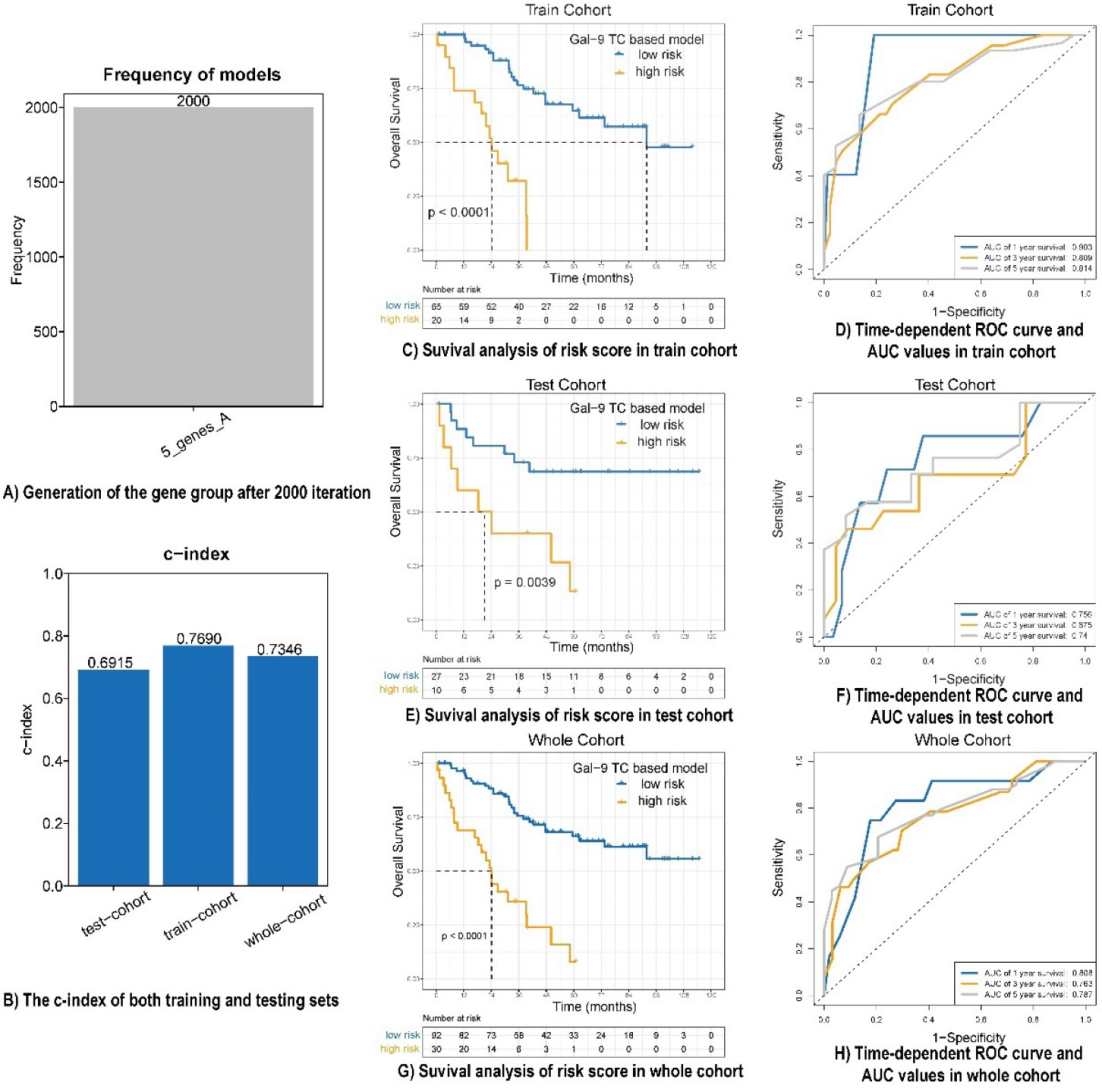
Prognostic performance of risk score model in LCNEC. **(A)** Generation of the gene group after 2000 iteration. **(B)** The c-index of both training, testing and whole cohort. **(C, E and G)** Survival analysis of risk score in train cohort, test cohort and whole cohort. **(D, F and H)** Time-dependent ROC curve and AUC values in train cohort, test cohort and whole cohort.

A univariate analysis was performed for all immune protein markers in the training cohort. After univariate COX analysis and log-rank test, five prognostic immune markers were selected (P<0.05). A Cox proportional hazards regression with tenfold cross-validation was run on the five immune protein markers to determine the best stratification model. Total 2000 iterations were performed, and the generated gene results are shown in Table S3. A gene model with five immune protein markers (CD4 on TILs, CD3 on TILs, Gal-9 on tumor cells, PD-1 on TILs, and PD-L1 on tumor cells) was with the all the frequencies of 2000 (Figure 2A). This gene model was the most suitable one, and was used to generate the immune marker-based risk score for LCNEC. Therefore, we utilized the five immune protein markers included in this model to construct the immune survival stratification risk score. The coefficient values of the five immune protein markers are listed in Table S4. All of the five immune protein markers, namely CD4 (HR = 0.57; 95% CI: 0.31-1.06, P = 0.047), CD3 (HR = 0.51; 95% CI: 0.29-0.9, P = 0.013), Gal-9 on tumor cells (HR=1.83; 95% CI: 1.01-3.31, P = 0.029), PD-1 (HR = 3.37; 95% CI: 1.86-6.09, P = 0.003), and PD-L1 on tumor cells (HR = 2.1; 95% CI: 1.19-3.72, P = 0.007) predicted survival in patients with LCNEC. Multivariate Cox regression analysis showed that Gal-9 on tumor cells was the independent prognostic feature in LCNEC (HR = 1.818; 95% CI: 1.022-3.234, P = 0.042) and the risk score consisted of five immune protein markers (HR = 4.49; 95% CI: 2.415-8.347, P <0.001) was significantly independently related to prognostic prediction (Table 2). The prognostic performance of the immune survival stratification was confirmed in the training cohort (c-index = 0.7690), test cohort (c-index = 0.6915), and the entire sample (c-index = 0.7346) (Figure 2B). In the training cohort, the risk score was associated with 1- (AUC: 0.903), 3- (AUC: 0.809), and 5-year (AUC: 0.814) survival. In the test cohort, the risk score was associated with 1- (AUC: 0.756), 3- (AUC: 0.675), and 5-year (AUC: 0:74) survival. In the entire cohort, the risk score was associated with 1- (AUC: 0.808), 3- (AUC: 0.763), and 5-year (AUC: 0.787) survival (Figure 2C-H).

### Galectin-9 RNA expression level in three kinds of lung neuroendocrine tumors

In the GEO Database, we examined mRNA expression of the LGALS9 gene (which encodes Gal-9) in carcinoid, (n = 24), LCNEC (n = 56), and SCLC (n = 21) cases. Carcinoid cases had significantly lower LGALS9 expression than LCNEC (P <0.001) and SCLC (P = 0.003) cases. Carcinoid had better disease-free survival (DFS) (P <0.001) and OS (P <0.001) than LCNEC and SCLC (Figure S2).

### Galectin-9 expression-related pathways

There were significant between-group differences across the whole 119 gene sets that were regulated differently. There were 106 upregulated gene sets in the high Gal-9 expression group that took up the majority of cases (n = 106, 89.08%). The top four high Gal-9 expression-related immune pathways (enrichment scores >0.75 and adjusted P <0.01) were: “graft-versus-host disease”, “allograft rejection”, “intestinal immune network for IgA production”, and “primary immunodeficiency” (Figure S3A). There was a close relationship and high degree of overlap between these pathways (Figure S3B). There were 12 genes overlapping in three of these pathways: CD28, CD40, CD86, HLA-DMA, HLA-DMB, HLA-DOA, HLA-DOB, HLA-DPA1, HLA-DPB1, HLA-DQB1, HLA-DRA, and HLA-DRB4. These 12 genes were also significantly enriched in cases with higher Gal-9 expression (Figure S3C, PPI enrichment p-value < 1.0e^-16^). STRING allowed us to visualize the highly significant protein-protein interaction between all 12 genes and LGALS9 (Figure S3D).

### Immune infiltration and LGALS9 expression

**Figure 3:**
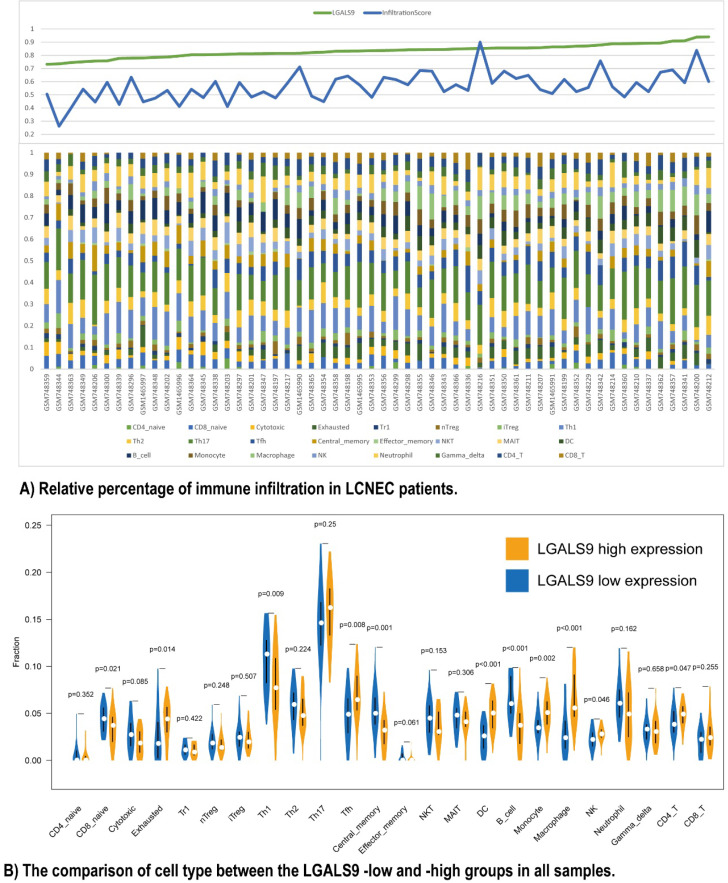
Relationship between immune cell proportions in LCNEC patients with LGALS9-high and LGALS9-low risk. **(A)** Relative percentage of immune infiltration in LCNEC patients. **(B)** The comparison of cell type between the LGALS9-low and -high groups in all samples.

Furthermore, we used ImmuCellAI to analyze the immune infiltration conditions between high- and low- LGALS9 expression groups. The infiltration score calculated from ImmuCellAI revealed the infiltrated immune cell number in tissues, which is positively associated with LGALS9 expression (Rho = 0.2903, *P* < 0.05, Figure 3A). The relative percentage of TILs varied in different samples (Figure 3B). CD4 T cells (*P* = 0.047), NK cells (*P* = 0.046), macrophage cells (*P* < 0.001), monocyte (*P* = 0.002), DC (*P* < 0.001), and Tfh cells (*P* = 0.008) have more infiltration in high-LGALS9 group, and B cell (*P* < 0.001), central memory cell (*P* = 0.001), Th1 (*P* = 0.009), naïve CD8+ T cell (*P* = 0.021) have less infiltration in high-LGALS9 group. However, interestingly, in cell quality estimation, the high-LGALS9 group has more the exhausted T cells infiltration (*P* = 0.014), besides, relatively less in cytotoxic T cell infiltration (*P* = 0.085). These results further confirmed the significance of LGALS9 on primary immunodeficiency as mentioned in GSEA results in the light of the immune infiltration in LCNEC.

### Galectin-9 expression-related biological processes

Given the quantity of immune cell infiltration could not comprehensively reveal the LGALS9's influence on primary immunodeficiency and the quantity alteration in exhausted T cells, cytotoxic T cell representing the ability of immune activity, for the sake of further understanding the immune cells quality alteration based on Gal-9 expression, we performed GSEA in GSE30219 dataset focusing on biological process. Among total 1786 gene sets between two LCNEC groups (adjusted *P* < 0.05), 1434 upregulated gene sets in the high Gal-9 expression group accounted for the largest proportion (1434/1786, 80.29%). Figure S4 demonstrated that the top 15 high Gal-9 expression-related biological process with enrichment scores > 0.85 and adjusted *P* < 0.01, and some interesting processes were as follows: “negative regulation of myeloid leukocyte mediated immunity”, “positive regulation of tolerance induction”, “negative regulation of cell killing”, “negative regulation of natural killer cell mediated immunity”, “negative regulation of natural killer cell mediated cytotoxicity”, which indicated the LGALS9's influence on primary immunodeficiency with worse or failure of immune activity.

### The five prognostic immune proteins marker-based survival stratification was associated with immune infiltration landscapes

**Figure 4:**
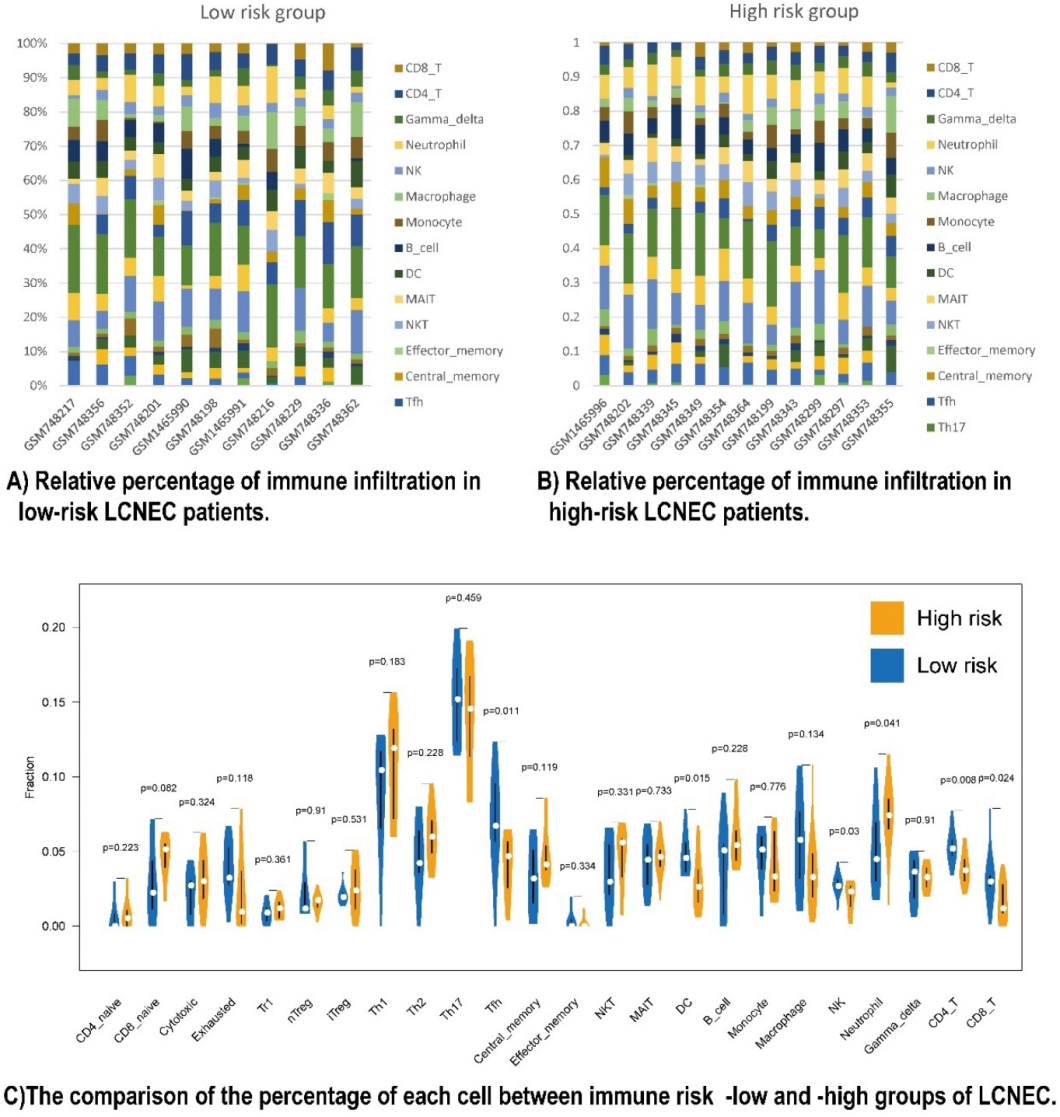
Relationship between immune cell proportions in LCNEC patients with high and low risk group. **(A and B)** Relative percentage of immune infiltration in low-risk and high-risk LCNEC patients. **(C)** The comparison of the percentage of each immune cell between immune risk- low and -high group of LCNEC.

To have a further understanding of the relationship among the five prognostic immune protein-coded genes (CD274, CD3E, CD4, LGALS9, and PDCD1), we found they were positively related among themselves in all 56 LCNEC tissues of GSE30219 (Figure S5A). To further study the mechanism of how our five prognostic immune proteins marker-based survival stratification influenced the prognosis of LCNEC, we analyzed the differences between 24 LCNEC tissues in terms of immune infiltration from GSE30219 which meets the criteria: 1. Low risk level has positive RNA expression of both CD4 and CD3E of which coefficient factors in our protein-based model are negative numbers, meanwhile, has no more than 2 of positive LGALS9, CD274, and PDCD1 of which coefficient factors in our protein-based model are positive numbers; 2. High risk level has both negative RNA expression of CD4 and CD3, meanwhile, at least 1 of positive LGALS9, CD274, and PDCD1 (Figure S6). The survival analysis showed LGALS9 RNA expression levels has not significantly difference on OS (*P* = 0.1649 Figure S5B) and DFS (*P* = 0.9604 Figure S5C), but based on the criteria above, the low-risk group (n = 11) and high-risk group (n = 13) in GSE30219 have a different trend meeting our rational on prognosis development, although without significant P value (Figure S5D, S5E). Two heatmaps demonstrated the detailed immune characteristics of 24 immune cells in different risk level patients with LCNEC (Figure 4A, 4B). CD8+ T (*P* = 0.024), CD4+ T (*P* = 0.008), NK cells (*P* = 0.03), DC (*P* = 0.015), and Tfh (*P* = 0.011) were scanter in high-risk group, but high-risk group was only filled with more neutrophils (*P* = 0.041) (Figure 4C). In conclusion, high-risk group seems like an immune-desert tumor with high level of LGALS9, PD-1, and PD-L1, which was consistent with the former result of LGALS9 inducing the positive regulation of immune tolerance. In conclusion, the high-risk group presented with immune-desert tumors characterized by high levels of LGALS9, PD-1, and PD-L1.

### Galectin-9 based immune survival risk level is associated with the sensitivity to immunotherapy

A subclass mapping algorithm was used to compare the RNA profiles of the high- and low- risk groups with another published dataset containing 47 cases of melanoma treated with immunotherapy. The results showed that the low-risk group was more likely to benefit from anti-PD1 immunotherapy (Bonferroni corrected *P* = 0.001), while the high-risk group was not sensitive to the immunotherapy (Figure S7). The low-risk group presented with immune-abundant tumors was sensitive to the immunotherapy.

### Galectin-9 based immune survival risk levels exert unique single cell pathological characteristics

**Figure 5:**
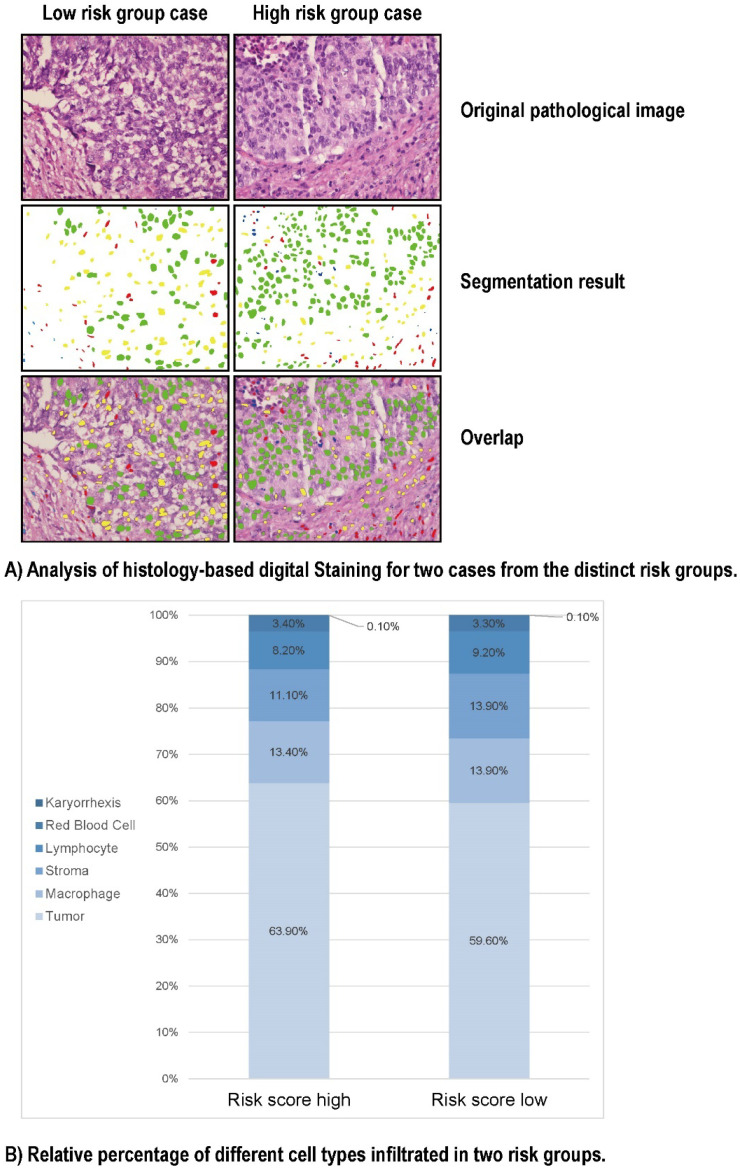
HD-Staining results for LCNEC between high risk and low risk groups. **(A)** Analysis of histology-based digital staining for two cases from the distinct risk groups. **(B)** Relative percentage of different cell types infiltrated in two risk groups.

To look into the relationship between image feature and the Gal-9 based immune survival risk levels, a total of 122 cases of LCNEC standard H&E-stained pathology images of our center was used in Histology-based Digital-Staining (HDS) to segmented various cells (Figure 5). A total of 108,369 cells were identified, including 65,764 tumor cells (60.7%), 14,279 stromal cells (13.2%), 9,663 lymphocytes (8.9%), 14,943 macrophages (13.8%), 117 karyorrhexis (0.1%), and 3,603 red blood cells (3.3%) (Table S5). The high-risk group had a higher proportion of tumor cells (63.9%) compared to the low-risk group (59.6%), while the low-risk group had more stromal cells (13.9% vs 11.1%) and lymphocytes (9.2% vs 8.2%). Eight cell nucleic imaging features were extracted, viz. area, convex area, eccentricity, filled area, major axis length, minor axis length, perimeter, and solidity. Higher tumor cell nucleic solidity was strongly related to high-risk group (OR = 3.048; 95% CI 1.513-6.138, P = 0.002), but interestingly, high-risk group usually has lower nucleic solidity of the stroma cell (OR = 0.253; 95% CI 0.097-0.665, P = 0.005) and the lymphocyte (OR = 0.081; 95% CI 0.017-0.392, P = 0.002), even lower nucleic minor axis length of the stroma cell (OR = 0.977; 95% CI 0.967-0.986, P < 0.001) as well as the lymphocyte (OR = 0.930; 95% CI 0.918-0.942, P < 0.001) (Table S6). The results above confirmed the former speculation of Gal-9 based immune survival stratification exerting the distinct immune type classification: high risk level group had an immune-desert feature although an activated tumor cell viability.

## Discussion

The expression of Gal-9 in tumor cells, but not TILs, was associated with prognosis. We established and validated a Gal-9-based immune risk model that predicted OS. The LCNEC immune infiltration profile of key genes provided insight into the important role of Gal-9 in LCNEC. Gal-9 based immune survival risk level is associated with the sensitivity to immunotherapy HDS provided a valuable new perspective, since it allowed us to detect the relationship between the TME and the immune marker-based risk model from pathological images.

Galectin-9 has a unique intestinal isoform and plays a significant role in regulating immune responses in the TME 10, 11. Gal-9 can impair the function of CD4-positive T cells and participate in the induction and differentiation of T regulator cells. The positive immunoregulatory function of Gal-9 has also been reported 16. Gal-9 is associated with the TME and immune infiltration in patients with SCLC, which has important clinical and research implications 17. It is expected that Gal-9 can be used as a therapeutic target for a wide variety of cancers, although its importance in LCNEC is less clear. In this study, Gal-9 expression on tumor cells was significantly correlated with pleural invasion. These clinical features are associated with poor outcomes in patients with LCNEC, suggesting that tumor Gal-9 expression is a marker of poor prognosis in LCNEC. However, prior data concerning the involvement of Gal-9 in pleural invasion is limited due to differences in tumor histology and sample sizes across prior studies 12, 18. We speculate that Gal-9 impairs the function of CD4-positive T cells, which, in turn, may promote tumor growth, migration, and invasion. Further studies are warranted to verify it and explore potential mechanisms which could explain this phenomenon.

The relationships between Gal-9 and other immune markers were specific to either tumor cell or TIL expression, which indicates the important role of the TME in tumorigenesis. On the one hand, a recent study showed that higher expression of Gal-9 inhibits the immune response through the TIM-3 pathway. CD8+ T cells co-expression of PD-1 and TIM-3 was associated with poorer OS in renal cell carcinoma 19. On the other hand, high Gal-9 expression was associated with a high level of stromal TILs and positive PD-L1 expression on tumor cells in breast cancer 20. Gal-9 may thus have a close connection with multiple immune markers.

In patients with LCNEC, positive Gal-9 expression on tumor cells was associated with OS, which remained significant in a subgroup analysis. Prior evidence concerning the relationship between Gal-9 expression and prognosis is inconsistent. Low expression of Gal-9, PD-L1 and CD8 is associated with a poor prognosis in hepatocellular carcinoma 21. The opposite results for PD-L1 and Gal-9 have been described for renal cell carcinoma 22. A meta-analysis showed that Gal-9 expression was associated with a favorable prognosis in both hepatocellular carcinoma and colorectal carcinoma, but also other characteristics, such as disease stage and the presence of lymph node metastasis 23. Low Gal-9 expression in tumor cells, and high Gal-9 expression in lymphocytes, have been associated with a poor prognosis in NSCLC 18. There are several reasons which could explain these conflicting findings. First, significant heterogeneity across different studies can lead to different results. For example, in lung cancer research, various pathological subtypes are often included 18. Second, Gal-9 is expressed in both tumor cells and lymphocytes, with variable clinical significance. Findings from total expression studies might therefore be misleading. The molecular risk score based on co-expression of Gal-9, CD4, CD3, PD-1 and PD-L1 on tumor cells was an accurate predictor of OS in both the entire cohort and the test cohort. Since LCNEC is rare, few prognostic models have combined Gal-9 with other immune markers.

The prognostic value of Gal-9 has been widely reported in lung cancer but without the consistent results 17, 18, 23. According to WHO 2015 classification, LCNEC and SCLC belong to the lung high-grade neuroendocrine tumors, and lung carcinoid tumors are the low-grade. Subsequent validation in GEO database found that LGALS9 was significantly elevated in high-grade tumors, and the prognosis of high-grade tumors was worse than that of low-grade tumors. Therefore, Gal-9 might be useful in other subtypes of lung cancer. Above results suggests that Gal-9 might be a characteristic of the malignancy marker of lung neuroendocrine tumors.

GSEA allowed us to characterize the top five high LGALS9-enriched pathways in LCNEC with 12 overlapping genes. The LGALS9-based network showed extensive interactions between LGALS9 and others including CD28, CD40, and CD86 and HLA family. These molecules are all expressed on immune cells and play a significant role in immune regulation. Gal-9 is associated with immunosuppression of the TME or induction of M2 polarization of macrophages 23, 24.

ImmuCellAI showed that LGALS9 was positively correlated with the infiltration score, and a specific landscape of 24 immune cell infiltration patterns was affected by Gal-9 expression. CD4, macrophages, DCs, and monocytes were elevated in the high-risk group, whereas central memory cells, Th1 cells, and B cells were elevated in the low-risk group high. The top 15 biological process related to LGALS9 included negative regulation of immunity. The T cell function cannot be fully restored by disruption of the PD-1/PD-L1 axis alone, indicating that additional negative regulatory pathways play a part such as TIM-3/Gal-9 promoting T ell exhaustion 9. However, Gal-9 expression is associated with dysfunctional T cell effector functions in the TME. Gal-9 is a marker of T cell exhaustion and a potential target for immunotherapy. Cytotoxic T cells play an important role in eliminating tumor cells. Our findings indicated that Gal-9 contributes to immune regulation by interacting with other immune cells. It is hypothesized that Gal-9 interacts with the 12 major immune pathway genes, leading to an increase in the number of immune-infiltrating cells, and a decrease in the quality of immune-infiltrating cells, including an increase in exhausted T-cell infiltration, and a decrease in cytotoxic T-cell infiltration in the high-risk group. Considering the relationship between worse OS and positive Gal-9 expression in tumor cells, patients with LCNEC might benefit from the incorporation of antibodies against Gal-9 plus other immune inhibitors. Gal-9-dominated TME signatures may be used to predict the response to immunotherapy, which relies on the activation of tumor-infiltrating immune cells, and inhibition of immune checkpoint pathways.

IHC showed that CD3 and CD4 were protective proteins, while PD1 and PD-L1 in tumor cells were negative proteins. We modified the immune risk score by combining LGALS9, CD3E, CD4, PDCD1 and CD274, which could provide a direction for immunotherapeutic targets in the future. These five prognostic immune protein-coded genes were positively related among themselves. There existed a considerable correlation among CD274, CD3E, CD4 and LGALS9. The PDCD1 and other prognostic immune protein-coded genes demonstrated a moderate correlation (Fig. S5A).

Low CD3 and CD4 were classified as high risk. The high-risk group had a poor prognosis, consistent with the characteristics of the high-risk group as immune-desert tumors: low CD4+ T cells and other lymphocytes. The high-risk group also had a lower immune infiltration score compared to the low-risk group. CD8+, CD4+, and NK cells were higher in the low-risk group, and neutrophils were higher in the high-risk group. As is known to all, CD8+, CD4+, NK cells are positively correlated with prognosis 25. T-cell suppression by neutrophils is implicated in a wide variety of tumors and are associated with poor survival in multiple cancers 26. A localized immunosuppressive niche is created for tumor survival by recruiting these cells to the tumor TME and priming them 24. Furthermore, by immunotherapy-sensitivity data mining, the low-risk group was more likely to respond to anti-PD1 immunotherapy, while the high-risk group showed no response to the immunotherapy. Taken together, our conclusions are in line with previous studies and revealed a novel combination of putative immunotherapeutic targets for LCNEC. Numerous studies have identified the potential therapeutic effects of Gal-9 in cancer. The complex interactions between tumor cells and the complex TME may affect the expression of Gal-9 and other immune molecules. Meanwhile, the correlation and mechanism of immunotherapy efficacy prediction deserve further exploration.

Our immune model was derived from IHC results and Gal-9 expression on tumor cells, but not TILs. Transcriptome profiling is traditionally done on bulk tumors, which contains a variety of cell types, such as tumor cells, stromal cells, and lymphocytes. A bulk tumor-based RNA sequencing approach may obscure or diminish the mRNA expression changes resulting from a single cell type or TME. Our findings are more precise and definitive at the protein level, dividing the tissue into tumor cells and stromal cells. For example, the expression of Gal-9 in tumor cells compared to lymphocytes has an opposing relationship with prognosis in patients with lung cancer 18. This also illustrates the complexity of tumor immune microenvironment interactions. We need to fully understand the mechanism of Gal-9 in LCNEC, balance the role of Gal-9 in immune promotion and immune suppression, and finally apply Gal-9 to tumor therapy.

The spatial distribution of cells can reflect cell growth types and spatial interactions between different cells 14. To further study the spatial distribution and interactions between different cell types, we analyzed all pathology images using an online tool 14. HDS was used to segment the nuclei of tumor, stromal cells, lymphocytes, macrophages, karyorrhexis, and red blood cells from standard pathology images. Compared with other image segmentation algorithms, HDS is faster, more convenient, and more accurate. The high-risk group did indeed have lower nucleic solidity of the stroma cell and the lymphocyte and a higher proportion of tumor cells based on HE stained sections in HDS. Gal-9-based immune prognostic models can affect the solidity of tumor cells as well as other stromal cells, with high nuclear karyorrhexis density indicating a higher proportion of tumor necrosis, leading to a poorer prognosis 27. Higher stromal nuclear density can better activate immune function and lead to a better prognosis. Integrating molecular data with HDS can in future be used to characterize the TME.

Our research differs from prior similar studies in several ways 28-29. The evaluation of multiple immune biomarkers in a single, large, homogeneous LCNEC cohort is an effective approach for developing a Gal-9-based immune risk model. The median follow-up time was longer, ensuring a higher accuracy and better performance of our model compared to others. Differential Gal-9 expression might result in variations in the tumor immune microenvironment and immune infiltration. Our study fully considered the transformation of the protein model in the application of transcriptome gene expression in the GEO dataset. Furthermore, the image-derived tumor and TME features obtain using HDS strengthened the relationship between Gal-9 risk model and different cell nucleus diversity, which confirmed the reduced immune infiltration in high-risk patients with LCNEC. Data from the pathology images has the potential to integrate spatial and molecular data to better characterize the TME. Additionally, our study added to the contribution of associating phenotype with the underlying tumor genotype, which has not been done in previous research.

Our study had several limitations, including a single-center and retrospective design. In future, prospective and multi-center studies are warranted. Meanwhile, we have a limited selection in terms of immune biomarkers. As we learn more about tumor immunology, other interesting molecules might be worthy of study.

## Conclusions

We used integrated pathological analysis to develop a Gal-9-based immune risk model to inform prognostication in patients with LCNEC. This model reflects that Gal-9 interacts with immune genes and pathways and impact immune infiltration from protein, RNA and morphological level. Furthermore, the low-risk group would probably benefit from anti-PD1 immunotherapy. Additionally, the immune risk score is closely related with the density of nuclei, which will be worth further exploration.

## Supplementary Material

Supplementary figures and tables 1,2,4,5,6.

Supplementary table 3.

## Figures and Tables

**Table 1:** Patients' characteristics (n=122)

Characteristic	N	(%)
**Gender**		
Female	10	8.2
Male	112	91.8
**Age**		
≤60 years old	37	30.3
>60 years old	85	69.7
**Drinker**		
No	55	45.1
Yes	67	54.9
**Smoker**		
No	12	9.8
Yes	110	90.2
**pT stage**		
1	28	23
2	55	45.1
3	22	18
4	17	13.9
**pN stage**		
0	88	72.1
1	14	11.5
2	18	14.8
3	2	1.6
**TNM stage**		
1	46	37.7
2	35	28.7
3	41	33.6
**Pathology**		
LCNEC	91	74.6
LCNEC with other type	31	25.4
**Differentiation**		
Poor	70	57.4
Well	52	42.6
**Pleurae invasion**		
No	54	44.3
Yes	68	55.7
**Vascular invasion**		
No	69	56.6
Yes	53	43.4
**Neuron invasion**		
No	110	90.2
Yes	12	9.8
**STAS**		
No	72	59
Yes	50	41
**Lymph node metastases**		
No	91	74.6
Yes	31	25.4
**Ki-67**		
<60%	63	51.6
>60%	59	48.4

STAS, spread through air space.

**Table 2:** Cox regression analysis

Variables	Univariate				Multivariate 1				Multivariate 2			
	p	HR	95%L	95%H	p	HR	95%L	95%H	p	HR	95%L	95%H
Gender (male vs female)	0.202	2.511	0.61	10.337								
Age (>60 vs ≤60)	**0.029**	2.233	1.086	4.59	**0.038**	2.176	1.044	4.534	0.069	1.992	0.948	4.186
Drinker (Yes vs No)	0.765	0.92	0.531	1.594								
Smoker (Yes vs No)	0.134	2.953	0.715	12.191								
**pT stage**												
1	0.3											
2	0.795	1.103	0.526	2.316								
3	0.232	1.669	0.72	3.868								
4	0.123	2.016	0.828	4.912								
**pN stage**												
0	**0.008**				**0.036**				**0.05**			
1	0.07	2.064	0.944	4.512	0.558	1.3	0.541	3.125	0.659	1.226	0.496	3.028
2	**0.022**	2.253	1.123	4.521	**0.247**	1.588	0.725	3.479	0.321	1.499	0.674	3.337
3	**0.008**	7.026	1.648	29.954	**0.006**	8.129	1.833	36.054	**0.007**	7.738	1.754	34.145
**TNM stage**												
1	**0.006**											
2	0.268	1.534	0.719	3.273								
3	**0.002**	2.938	1.487	5.803								
Pathology (LCNEC+other vs LCNEC)	0.579	1.191	0.643	2.205								
Differentiation (well vs poor)	0.336	1.311	0.755	2.275								
Pleurae invasion (Yes vs No)	0.282	1.372	0.771	2.439								
Vascular invasion (Yes vs No)	**0.032**	1.836	1.052	3.203	0.105	1.737	0.89	3.388	0.449	1.311	0.651	2.638
Neuron invasion (Yes vs No)	0.402	1.488	0.587	3.772								
STAS (Yes vs No)	0.392	1.283	0.725	2.269								
Lymph node metastases (Yes vs No)	**0.028**	1.91	1.072	3.405								
Ki-67 (>60% vs <60%)	0.682	1.122	0.647	1.945								
Gal-9 TC (positive vs negative)	**0.032**	1.832	1.054	3.186	**0.042**	1.818	1.022	3.234				
Risk score (high vs low)	**<0.001**	5.045	2.804	9.076					**<0.001**	4.49	2.415	8.347

STAS, spread through air space; Gal-9 TC, galectin-9 on tumor cells. Statistically significant data were marked with bold and underline.

## Abbreviations

AUC: Area under the receiver operating curve
CD: Cluster of differentiation
DC: Dendritic cell
DFS: Disease-free survival
Gal-9: Galectin-9
GEO: Gene expression omnibus
HR: Hazard ratio
HDS: Histology-based digital staining
ICI: Immune checkpoint inhibitors
ImmuCellAI: Immune Cell Abundance Identifier
LCNEC: Large cell neuroendocrine tumors
NK: Natural killer
OR: Odds ratio
OS: Overall survival
SCLC: Small cell lung cancer
TIL: Tumor infiltrating lymphocyte
TME: Tumor micro-environment
