# Supplementary material for: Integrated pathological analysis to develop a Gal-9 based immune survival stratification to predict the outcome of lung large cell neuroendocrine carcinoma and its usefulness in immunotherapy
Source: Int J Biol Sci. 2022 Sep 25;18(15):5913–27. doi: 10.7150/ijbs.76936 (PMC9576518; doi:10.7150/ijbs.76936)
Supplement: Supplementary file 1 — Supplementary figures and tables 1,2,4,5,6. [file ijbsv18p5913s1.pdf]

Supplementary Figure

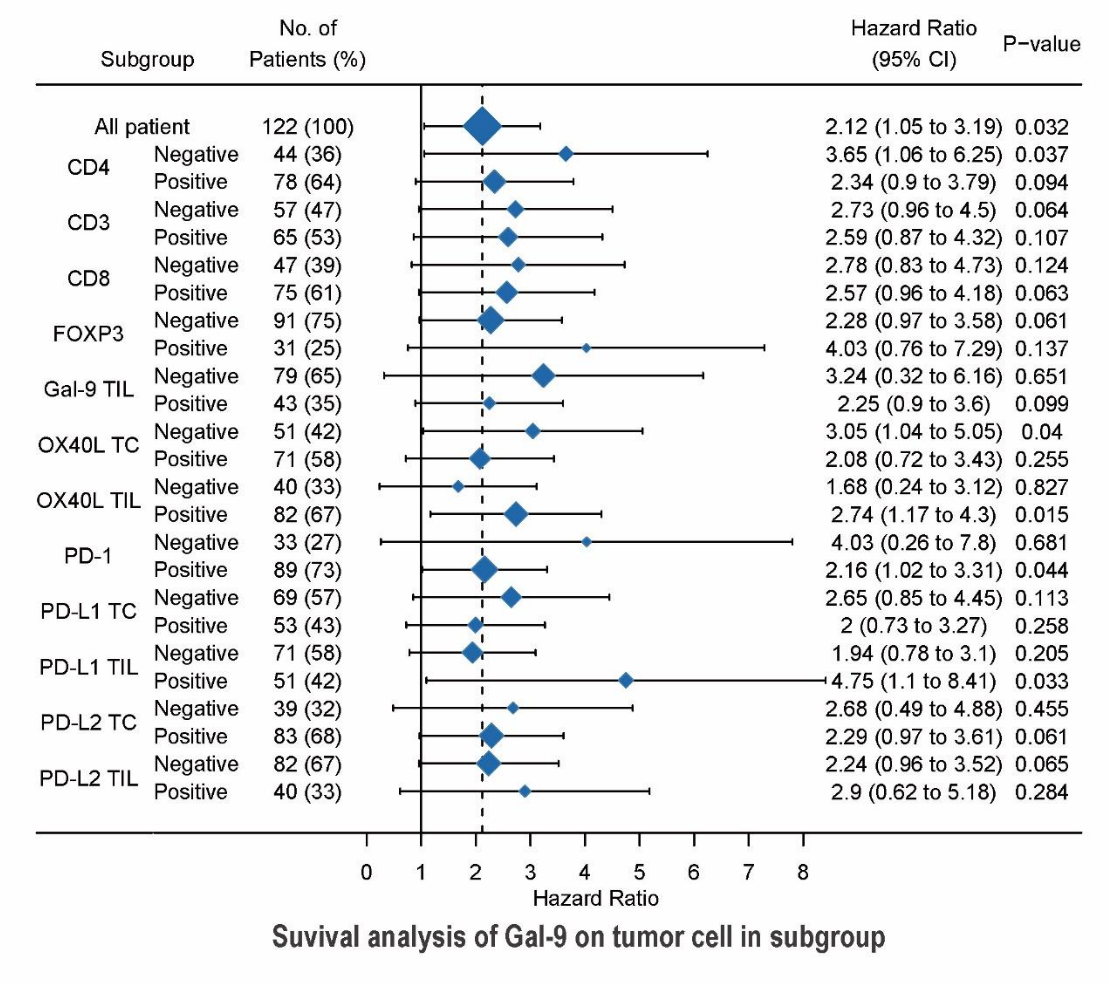

Fig S1. Survival analysis of Gal-9 on tumor cells in subgroup

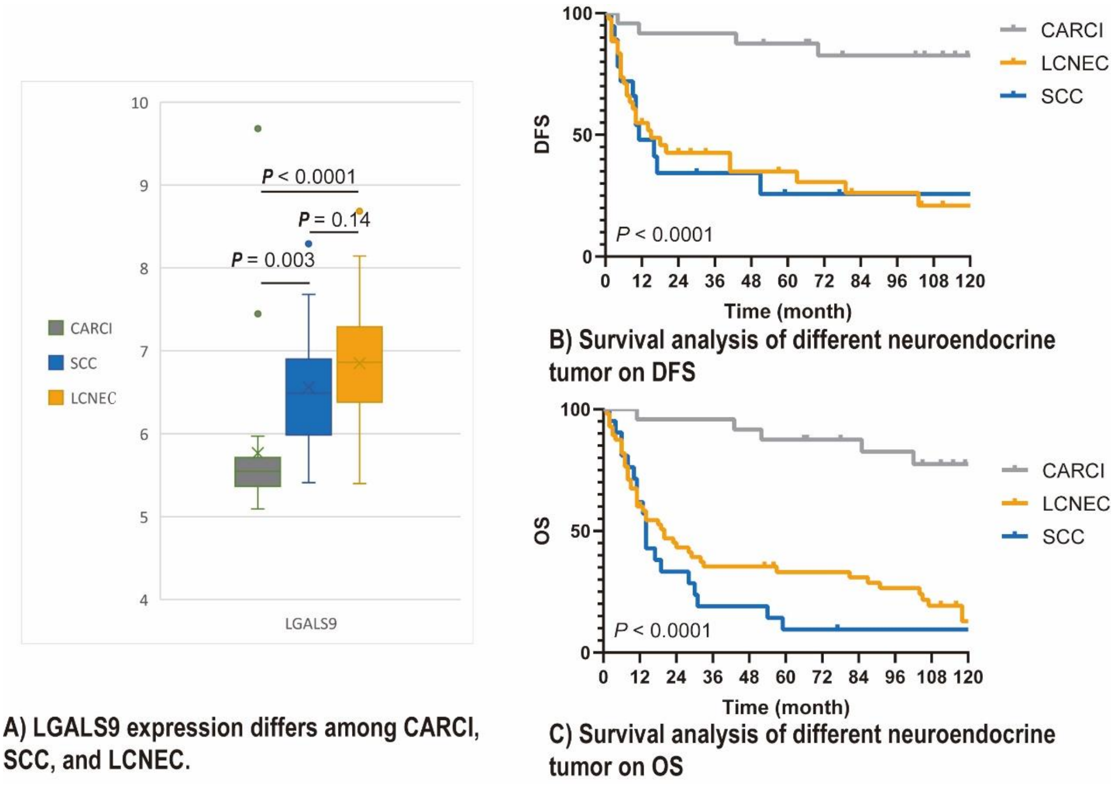

Fig S2. Validation of LGALS9 expression and survival analysis in pulmonary neuroendocrine tumor. (A) LGALS9 expression differs among CARCI, SCC and LCNEC. (B) Survival analysis of different neuroendocrine tumor on DFS. (C) Survival analysis of different neuroendocrine tumor on OS.

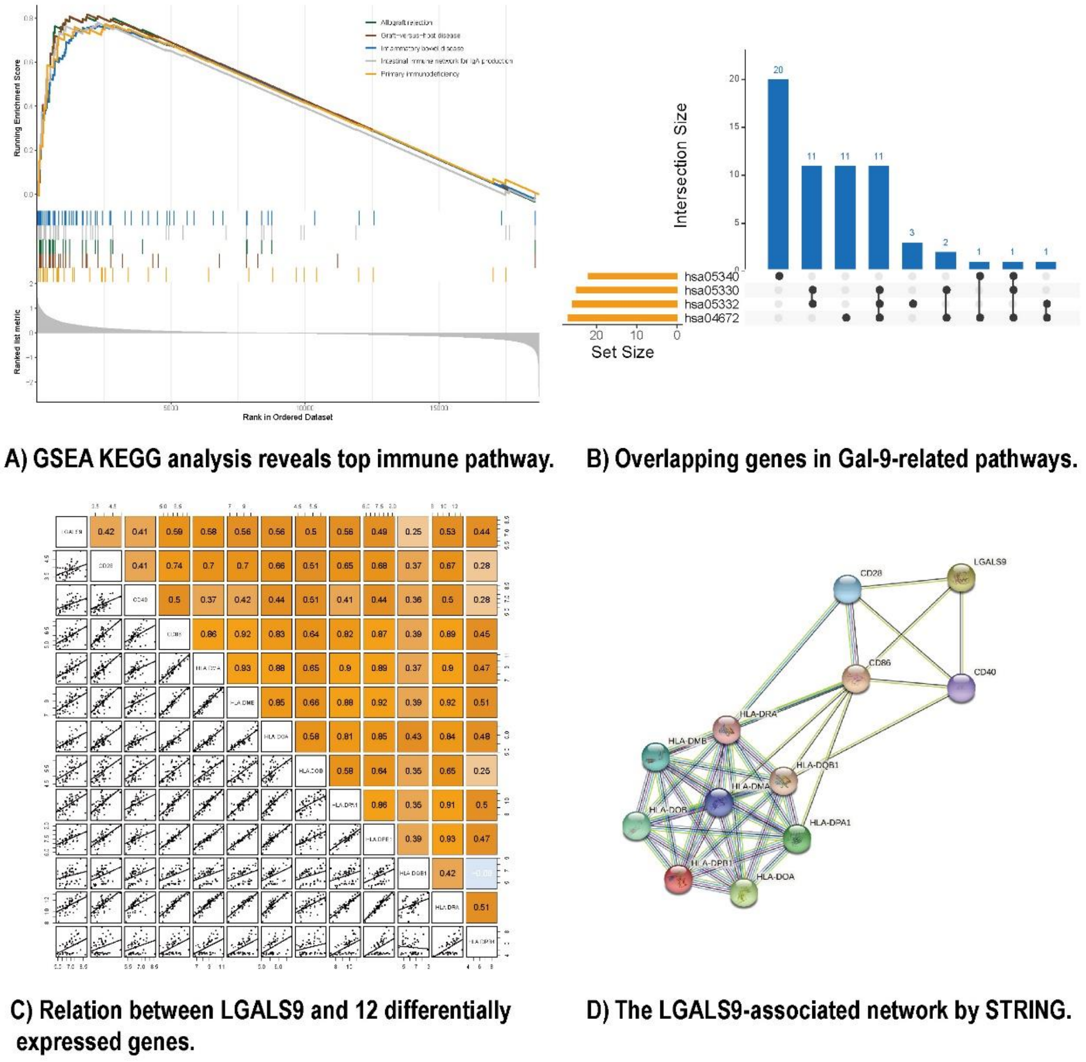

A) GSEA KEGG analysis reveals top immune pathway.

B) Overlapping genes in Gal-9-related pathways.

C) Relation between LGALS9 and 12 differentially expressed genes.

D) The LGALS9-associated network by STRING.

Fig S3. Analysis of top Gal-9-related pathways in LCNEC. (A) GSEA and KEGG analysis reveals top Gal-9 related immune pathway; (B) Overlapping genes in Gal-9-related pathways; (C) Relation between LGALS9 and 12 differentially expressed genes; (D) The LGALS9-associated network by STRING.

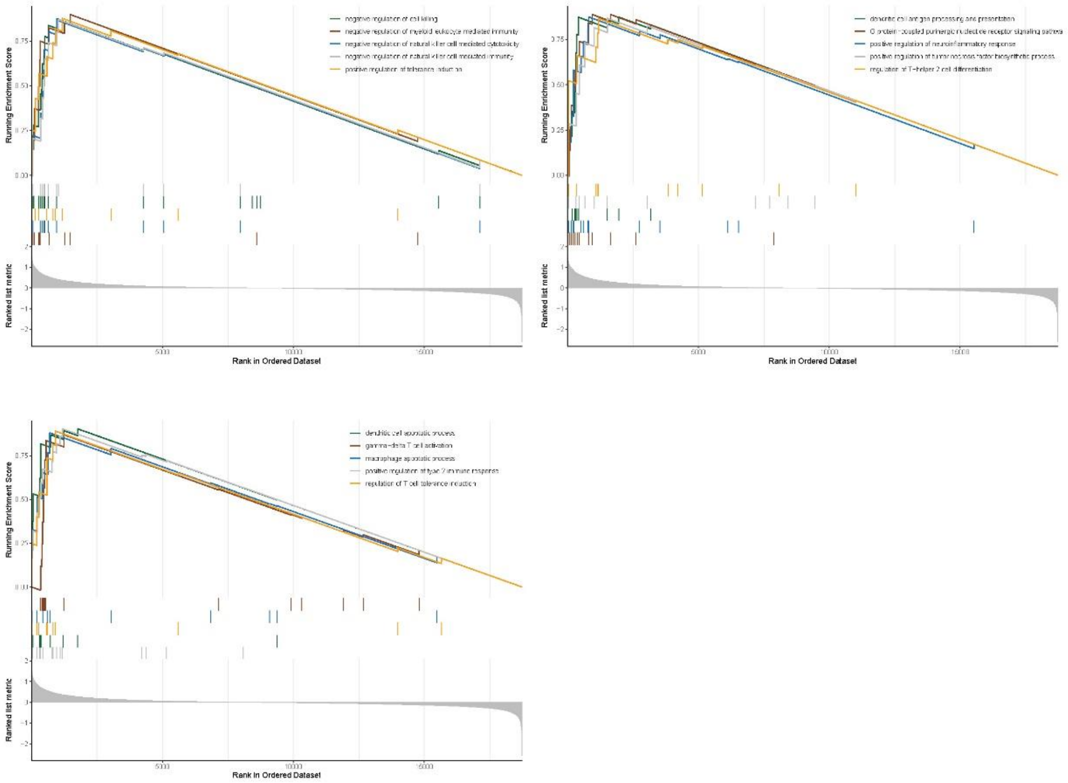

GSEA GO analysis reveals top15 biological process related to LGALS9 in LCNE.

Fig S4. GSEA and GO analysis reveals top 15 biological process related to LGALS9 in LCNEC.

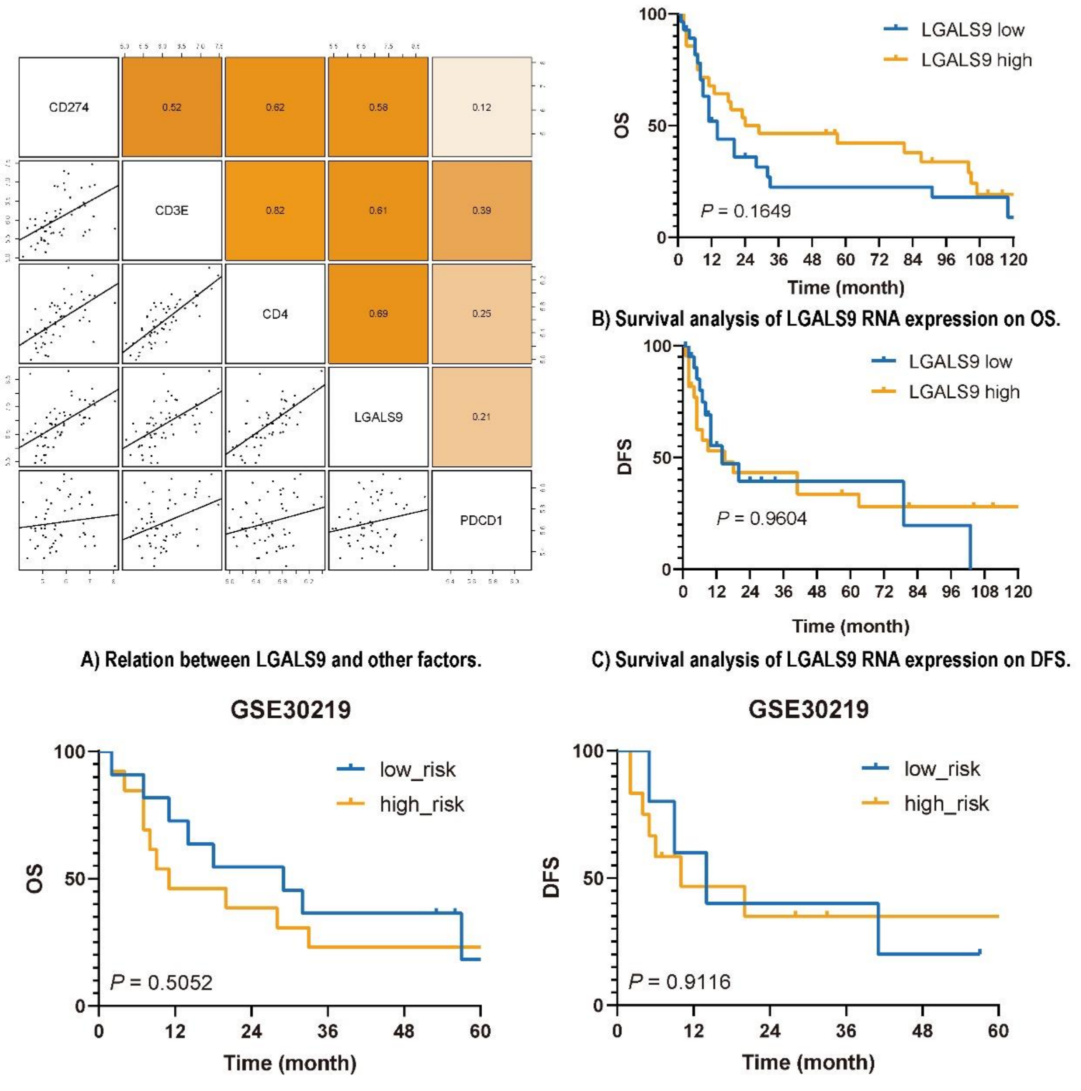

A) Relation between LGALS9 and other factors.

B) Survival analysis of LGALS9 RNA expression on OS.

C) Survival analysis of LGALS9 RNA expression on DFS.

D) Survival analysis of immune risk score on OS.

E) Survival analysis of immune risk score on DFS.

Fig S5. Clinical value of LGALS9 and immune risk score in GSE30219. (A) Relationship between LGALS9 and other factors, including CD274, CD3E, CD4 and PDCD1. (B) Survival analysis by LGALS9 RNA expression on OS; (C) Survival analysis by LGALS9 RNA expression on DFS; (D) Survival analysis by immune risk score on OS; (E) Survival analysis by immune risk score on DFS.

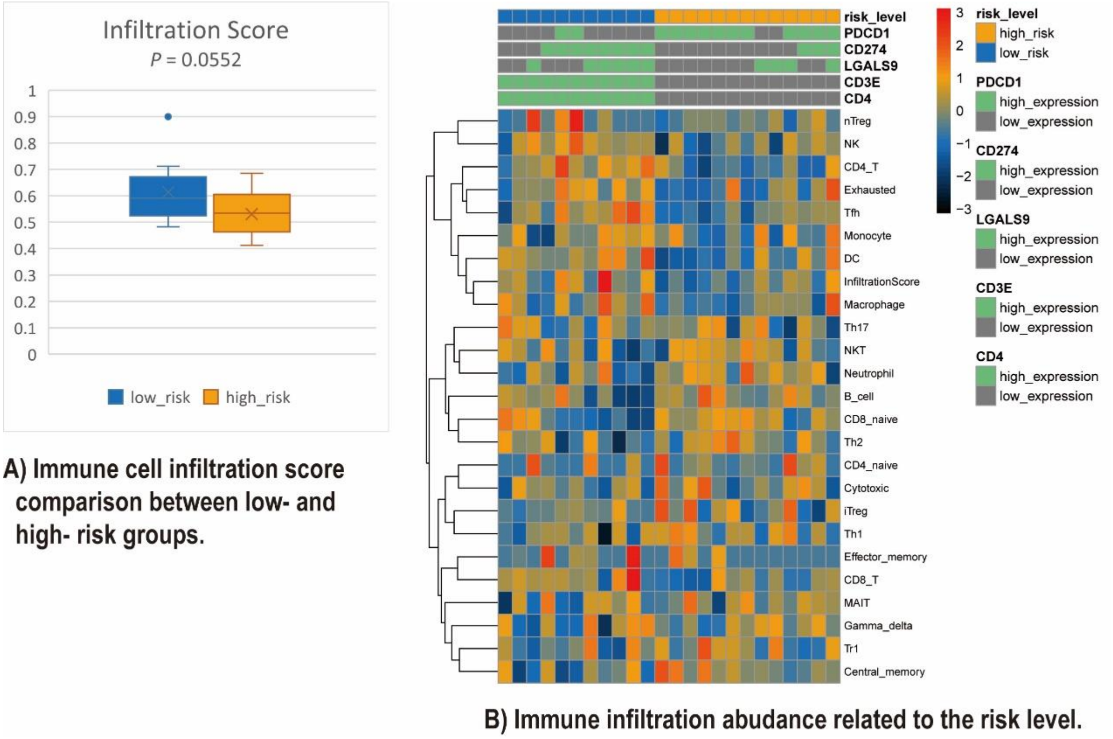

Fig S6. Immune cell infiltration score and abundance between low- and high- risk groups. (A) Immune cell infiltration score comparison between low- and high- risk groups. (B) Immune infiltration abundance related to the risk level.

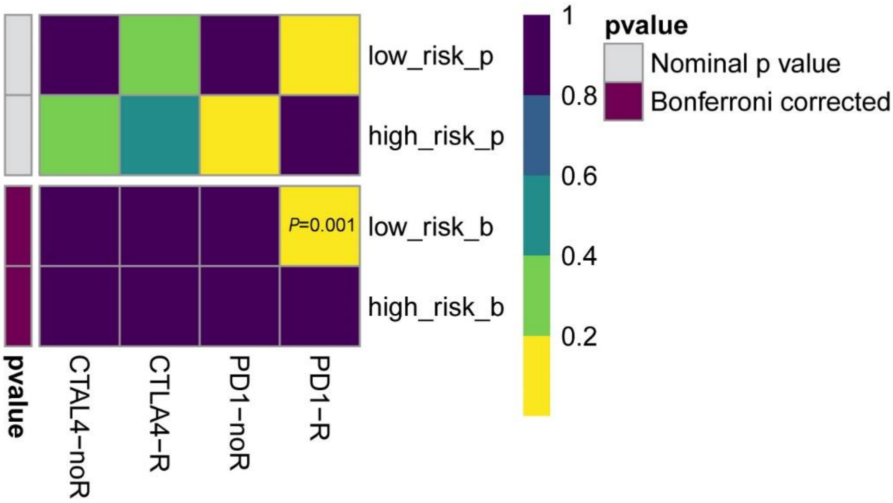

Fig S7. Differential putative immunotherapeutic response. The low-risk group has a promising response to anti-PD1therapy (Bonferroni corrected P=0.001).

Supplementary Table

Table S1 Relationship between galectin-9 (Gal-9) and clinical factors

	Gal-9 expression on tumor cells			Gal-9 expression on TILs		
Variables	Negative	Positive	P value	Negative	Positive	P value
Gender			0.743			0.662
Female	6 (60.0%)	4 (40.0%)		5 (50.0%)	5 (50.0%)	
Male	73 (65.2%)	39 (34.8%)		48 (42.9%)	64 (57.1%)	
Age			0.4			0.713
≤60 years old	26 (70.3%)	11 (29.7%)		17 (45.9%)	20 (54.1%)	
>60 years old	53 (62.4%)	32 (37.6%)		36 (42.4%)	49 (57.6%)	
Drinker			0.079			0.254
No	31 (56.4%)	24 (43.6%)		27 (49.1%)	28 (50.9%)	
Yes	48 (71.6%)	19 (28.4%)		26 (38.8%)	41 (61.2%)	
Smoker			0.624			0.896
No	7 (58.3%)	5 (41.7%)		5 (41.7%)	7 (58.3%)	
Yes	72 (65.5%)	38 (34.5%)		48 (43.6%)	62 (56.4%)	
pT stage			0.733			0.482
1	19 (67.9%)	9 (32.1%)		9 (32.1%)	19 (67.9%)	
2	36 (65.5%)	19 (34.5%)		24 (43.6%)	31 (56.4%)	
3	15 (68.2%)	7 (31.8%)		11 (50.0%)	11 (50.0%)	
4	9 (52.9%)	8 (47.1%)		9 (52.9%)	8 (47.1%)	
pN stage			0.51			0.614
0	59 (67.0%)	29 (33.0%)		41 (46.6%)	47 (54.4%)	
1	8 (57.1%)	6 (42.9%)		4 (28.6%)	10 (71.4%)	
2	10 (55.6%)	8 (44.4%)		7 (38.9%)	11 (61.1%)	
3	2 (100%)	0 (0%)		1 (50.0%)	1 (50.0%)	
TNM stage			0.31			0.671
1	33 (71.7%)	13 (28.3%)		21 (45.7%)	25 (54.3%)	
2	23 (65.7%)	12 (34.3%)		13 (37.1%)	22 (62.9%)	
3	23 (56.1%)	18 (43.9%)		19 (46.3%)	22 (53.7%)	
Pathology			0.974			0.823
LCC	59 (64.8%)	32 (35.2%)		39 (42.9%)	52 (57.1%)	
LCC with other type	20 (64.5%)	11 (35.5%)		14 (45.2%)	17 (54.8%)	
Differentiation			0.306			0.88
Poor	48 (68.6%)	22 (31.4%)		30 (42.9%)	40 (57.1%)	
Well	31 (59.6%)	21 (40.4%)		23 (44.2%)	29 (55.8%)	
Pleurae invasion			**0.007**			0.866
No	42 (77.8%)	12 (22.2%)		23 (42.6%)	31 (57.4%)	
Yes	37 (54.4%)	31 (45.6%)		30 (44.1%)	38 (55.9%)	
Vascular invasion			0.375			0.993
No	47 (68.1%)	22 (31.9%)		30 (43.5%)	39 (56.5%)	
Yes	32 (60.4%)	21 (39.6%)		23 (43.4%)	30 (56.6%)	
Neuron invasion			0.884			0.087
No	71 (64.5%)	39 (35.5%)		45 (40.9%)	65 (59.1%)	
Yes	8 (66.7%)	4 (33.3%)		8 (66.7%)	4 (33.3%)	
STAS			0.532			0.223

No	45 (62.5%)	27 (37.5%)		28 (38.9%)	44 (61.1%)	
Yes	34 (68.0%)	16 (32.0%)		25 (50.0%)	25 (50.0%)	
Lymph node metastases			0.367			0.301
No	61 (67.0%)	30 (33.0%)		42 (46.2%)	49 (53.8%)	
Yes	18 (58.1%)	13 (41.9%)		11 (35.5%)	20 (64.5%)	
Ki-67			0.496			0.11
<60%	39 (61.9%)	24 (38.1%)		23 (36.5%)	40 (63.5%)	
>60%	40 (67.8%)	19 (32.2%)		30 (50.8%)	29 (49.2%)	

Table S2 Relationship between galectin-9 (Gal-9) and other checkpoints

	Gal-9 expression on tumor cells			Gal-9 expression on TILs		
Variables	Negative	Positive	P value	Negative	Positive	P value
Gal-9 on tumor cells						**<u><0.001</u>**
Negative	/	/	/	48 (60.8%)	31 (39.2%)	
Positive				5 (11.6%)	38 (88.4%)	
CD4			**<u>0.03</u>**			**<u>0.025</u>**
Negative	34 (77.3%)	10 (22.7%)		25 (56.8%)	19 (43.2%)	
Positive	45 (57.7%)	33 (42.3%)		28 (35.9%)	50 (64.1%)	
CD3			0.24			0.236
Negative	40 (70.2%)	17 (29.8%)		28 (49.1%)	29 (50.9%)	
Positive	39 (60.0%)	26 (40.0%)		25 (38.5%)	40 (61.5%)	
CD8			**<u>0.03</u>**			**<u><0.001</u>**
Negative	36 (76.6%)	11 (23.4%)		29 (61.7%)	18 (38.3%)	
Positive	43 (57.3%)	32 (42.7%)		24 (32.0%)	51 (68.0%)	
FOXP3			0.076			**<u>0.022</u>**
Negative	63 (69.2%)	28 (30.8%)		45 (49.5%)	46 (50.5%)	
Positive	16 (51.6%)	15 (48.4%)		8 (25.8%)	23 (74.2%)	
OX40L_TC			0.992			**<u>0.03</u>**
Negative	33 (64.7%)	18 (35.3%)		28 (54.9%)	23 (45.1%)	
Positive	46 (64.8%)	25 (35.2%)		25 (35.2%)	46 (64.8%)	
OX40L_TIL			0.098			**<u><0.001</u>**
Negative	30 (75.0%)	10 (25.0%)		27 (67.5%)	13 (32.5%)	
Positive	49 (59.8%)	33 (40.2%)		26 (31.7%)	56 (68.3%)	
PD 1			0.121			**<u>0.002</u>**
Negative	25 (75.8%)	8 (24.2%)		22 (66.7%)	11 (33.3%)	
Positive	54 (60.7%)	35 (39.3%)		31 (34.8%)	58 (65.2%)	
PD L1_TC			0.099			**<u>0.026</u>**
Negative	49 (71.0%)	20 (29.0%)		36 (52.2%)	33 (47.8%)	
Positive	30 (56.6%)	23 (43.4%)		17 (32.1%)	36 (67.9%)	
PD L1_TIL			0.245			0.425
Negative	49 (69.0%)	22 (31.0%)		33 (46.5%)	38 (53.5%)	
Positive	30 (58.8%)	21 (41.2%)		20 (39.2%)	31 (60.8%)	
PD L2_TC			**<u>0.02</u>**			**<u>0.048</u>**
Negative	31 (79.5%)	8 (20.5%)		22 (56.4%)	17 (43.6%)	
Positive	48 (57.8%)	35 (42.2%)		31 (37.3%)	52 (62.7%)	
PD L2_TIL			0.968			0.189
Negative	53 (64.6%)	29 (35.4%)		39 (47.6%)	43 (52.4%)	
Positive	26 (65.0%)	14 (35.0%)		14 (35.0%)	26 (65.0%)	

Table S4. The coefficient factors of five protein markers in optimal model.

Protein	coefficient	Gene Symbol
CD4	-0.849835	CD4
CD3	-0.109617	CD3E
Gal.9_TC	0.8515026	LGALS9
PD1	2.2025665	PDCD1
PD L1_TC	0.7099509	CD274

Table S5. Characteristics of different cells

	Tumor cell			
	HIGH risk	LOW risk	p test	SMD
Tumor cell n	18094	47670		
area (mean (SD))	506.29 (294.25)	502.49 (283.05)	0.128	0.013
convex_area (mean (SD))	524.59 (305.02)	521.17 (294.16)	0.187	0.011
eccentricity (mean (SD))	0.70 (0.15)	0.70 (0.15)	0.016	0.021
filled_area (mean (SD))	506.32 (294.26)	502.53 (283.13)	0.129	0.013
major_axis_length (mean (SD))	30.25 (9.32)	30.35 (9.18)	0.212	0.011
minor_axis_length (mean (SD))	20.28 (6.50)	20.20 (6.25)	0.12	0.013
perimeter (mean (SD))	83.24 (25.50)	83.33 (24.85)	0.676	0.004
solidity (mean (SD))	0.97 (0.02)	0.96 (0.03)	0.002	0.027
	Stroma cell			
	HIGH risk	LOW risk	p test	SMD
Tumor cell n	3140	11139		
area (mean (SD))	209.17 (146.76)	220.71 (153.56)	<0.001	0.077
convex_area (mean (SD))	223.53 (160.36)	234.83 (165.92)	0.001	0.069
eccentricity (mean (SD))	0.81 (0.15)	0.80 (0.15)	0.01	0.052
filled_area (mean (SD))	209.18 (146.79)	220.72 (153.63)	<0.001	0.077
major_axis_length (mean (SD))	23.02 (9.51)	23.28 (9.53)	0.184	0.027
minor_axis_length (mean (SD))	11.27 (4.00)	11.69 (4.21)	<0.001	0.103
perimeter (mean (SD))	56.62 (21.75)	57.66 (22.06)	0.019	0.047
solidity (mean (SD))	0.94 (0.04)	0.95 (0.04)	0.001	0.068
	Red Blood Cell			
	HIGH	LOW	p test	SMD
Tumor cell n	961	2642		
area (mean (SD))	187.47 (185.97)	194.38 (239.66)	0.418	0.032
convex_area (mean (SD))	197.19 (204.09)	203.95 (254.45)	0.458	0.029
eccentricity (mean (SD))	0.70 (0.16)	0.69 (0.16)	0.138	0.056
filled_area (mean (SD))	187.51 (186.11)	194.39 (239.66)	0.421	0.032
major_axis_length (mean (SD))	18.54 (7.87)	18.41 (8.07)	0.673	0.016
minor_axis_length (mean (SD))	11.96 (4.30)	12.12 (4.74)	0.335	0.037
perimeter (mean (SD))	49.15 (21.15)	49.11 (22.05)	0.956	0.002
solidity (mean (SD))	0.96 (0.03)	0.96 (0.03)	0.943	0.003
	Marcrophage			
	HIGH	LOW	p test	SMD
Tumor cell n	3782	11161		
area (mean (SD))	296.22 (171.17)	307.21 (180.22)	0.001	0.063
convex_area (mean (SD))	308.68 (179.42)	320.34 (189.42)	0.001	0.063
eccentricity (mean (SD))	0.70 (0.16)	0.69 (0.16)	0.095	0.031
filled_area (mean (SD))	296.25 (171.22)	307.23 (180.26)	0.001	0.062
major_axis_length (mean (SD))	23.35 (7.22)	23.66 (7.53)	0.025	0.043
minor_axis_length (mean (SD))	15.54 (4.68)	15.85 (4.82)	0.001	0.066
perimeter (mean (SD))	63.37 (19.39)	64.49 (20.26)	0.003	0.056
solidity (mean (SD))	0.96 (0.03)	0.96 (0.03)	0.59	0.01
	Lymphocyte			
	HIGH	LOW	p test	SMD
Tumor cell n	2325	7338		
area (mean (SD))	115.88 (84.39)	135.25 (89.79)	<0.001	0.222

convex_area (mean (SD))	120.82 (89.11)	140.24 (93.21)	<0.001	0.213
eccentricity (mean (SD))	0.68 (0.17)	0.67 (0.16)	<0.001	0.083
filled_area (mean (SD))	115.88 (84.42)	135.26 (89.79)	<0.001	0.222
major_axis_length (mean (SD))	14.17 (5.57)	15.15 (5.53)	<0.001	0.177
minor_axis_length (mean (SD))	9.52 (3.58)	10.48 (3.77)	<0.001	0.261
perimeter (mean (SD))	37.19 (15.15)	40.34 (15.02)	<0.001	0.208
solidity (mean (SD))	0.96 (0.03)	0.97 (0.03)	0.003	0.067

	Karyorrhexis				
	HIGH	LOW	p	test	SMD
Tumor cell n	27	90			
area (mean (SD))	111.96 (79.38)	131.66 (89.44)	0.306		0.233
convex_area (mean (SD))	117.15 (83.17)	138.42 (96.62)	0.303		0.236
eccentricity (mean (SD))	0.70 (0.18)	0.73 (0.15)	0.45		0.158
filled_area (mean (SD))	111.96 (79.38)	131.66 (89.44)	0.306		0.233
major_axis_length (mean (SD))	14.69 (5.34)	15.86 (6.34)	0.386		0.2
minor_axis_length (mean (SD))	9.15 (4.03)	9.87 (3.75)	0.391		0.185
perimeter (mean (SD))	37.76 (14.28)	41.08 (16.66)	0.351		0.214
solidity (mean (SD))	0.96 (0.02)	0.96 (0.03)	0.737		0.076

Table S6. Forward LR Multi-logistics analysis to investigate the relationship between cell imaging characteristics and risk level.

Cell type		Tumor cell	Stroma cell		Macrophage	Lymphocyte	
Image characteristics		solidity	Minor _axis_ length	solidity	minor_ axis_ length	minor_ axis_ length	solidity
P		<u>**0.002**</u>	<u>**0.000**</u>	<u>**0.005**</u>	<u>**0.001**</u>	<u>**0.000**</u>	<u>**0.002**</u>
OR		3.048	0.977	0.253	0.986	0.930	0.081
OR 95%CI	low	1.513	0.967	0.097	0.979	0.918	0.017
	high	6.138	0.986	0.665	0.994	0.942	0.392
